# The Effect of Transportation on Puppy Welfare from Commercial Breeding Kennels to a Distributor

**DOI:** 10.3390/ani12233379

**Published:** 2022-12-01

**Authors:** Aynsley C. Romaniuk, Alessia Diana, Shanis Barnard, Jennifer E. Weller, Uri Baqueiro Espinosa, Sriveny Dangoudoubiyam, Traci Shreyer, Gareth Arnott, Candace Croney

**Affiliations:** 1 Department of Comparative Pathobiology, Purdue University, 725 Harrison Street, West Lafayette, IN 47906, USA; 2 Animal Welfare Unit—Livestock Production Sciences Branch, Sustainable Agri-Food Sciences Division, Agri-Food and Biosciences Institute, Hillsborough BT26 6DR, UK; 3 School of Biological Sciences, Queen’s University Belfast, Belfast BT9 5DL, UK

**Keywords:** puppy, transportation, commercial breeding kennel, behavior, cortisol, IgA, parasite, welfare

## Abstract

**Simple Summary:**

Puppies from commercial breeding kennels (CBKs) are transported by ground from their facilities of origin to a distributor at approximately 8 weeks of age, which is a sensitive period in dogs’ early development. Experiencing high levels of fear and stress during this transition potentially jeopardizes their short- and long-term welfare. However, no research has explored the effect of transportation on puppy welfare. Therefore, we tested 383 puppies at 12 CBKs and again approximately 48 h after transportation to a distributor. We measured puppies’ behaviors in isolation and during a stranger-approach test, and conducted a physical health assessment. Feces were also collected from each litter and from one focal puppy per litter for insight into their stress response, immune function, and presence of intestinal parasites. Overall, puppies were physically healthy before and after transportation. However, behavioral and physiological findings suggest puppies experienced an increase in distress after transportation. Results also suggest that puppies may have solicited more contact from an unfamiliar person as a coping strategy following an acute stressor after transportation. Future studies should investigate risk factors associated with transportation protocols and identify interventions to help optimize puppy welfare during this transition.

**Abstract:**

Many puppies from commercial breeding kennels (CBKs) are transported by ground from their kennels of origin to a distributor. This experience may elicit fear and stress during a sensitive developmental period, which may in turn negatively impact the puppies’ short- and long-term welfare. This study aimed to measure short-term effects of transportation on puppy welfare metrics. Eight-week-old puppies (*n* = 383) from 12 CBKs were tested at their kennels (pre-trans) and ~48 h after arriving at a distributor (post-trans). At each location, puppies underwent an isolation test, a stranger-approach test, and a physical health assessment. Behavioral responses to testing were scored from videos. Fecal glucocorticoid metabolites (FGM), fecal secretory immunoglobulin A (sIgA), and presence of intestinal parasites were also analyzed. Linear mixed-effects models identified decreased exploration (*p* < 0.001), and increased locomotion (*p* < 0.001) and escape attempts (*p* = 0.001) during the post-trans isolation test. Increased affiliative behavior (*p* < 0.001), FGM (*p* < 0.001) and sIgA (*p* = 0.014) were also observed post-trans. Findings support good physical health both pre- and post-trans, while behavioral and physiological changes suggest increased puppy distress post-trans. Higher post-transport affiliative behavior may indicate that puppies sought social support as a coping strategy after experiencing transport-related distress. Future studies should explore the efficacy of transportation-related interventions to mitigate puppy distress.

## 1. Introduction

Transportation may elicit distress in many species [1,2,3]. Factors such as unloading and loading of animals, temperature and humidity of the transport environment, presence of motion sickness, quality of human-caretaker interactions, and space allowance can influence the degree of distress an animal may experience [4,5,6]. Furthermore, not only must animals endure the stressors associated with transportation itself, but many will experience separation from their conspecifics, unpredictable stimuli, and a novel environment upon arrival to their destination. This accumulation of stress may lead to immunosuppression and increased susceptibility to disease, negative affective states, and fewer displays of species-specific natural behaviors, resulting in poor animal welfare [4,7]. Consequently, stakeholders could risk decreased economic benefits due to sick animals and increased health concerns due to zoonotic disease susceptibility [4,5].

Most literature exploring the effects of transportation on animal welfare is on livestock species, while the research on companion animals is scarce. Of those studies published in dogs, owner-reports indicated that 23.8–43.6% of pet dogs in Italy had travel-related issues consisting of panting, restlessness, vocalization, and vomiting while being transported by car [6,8]. Mariti et al. [8] suggested that these issues may partially be due to early life experiences, as dogs which were accustomed to car transportation early in life were at a decreased risk of developing travel-related issues later on. Dogs are likely to struggle with transportation in adulthood if there is a lack of positive exposure to it during the socialization period of development (approximately 3 to 12–14 weeks of age), which is when exposure to certain stimuli and environments can elicit long-term and irreversible effects on later behavior [9,10]. This is especially true if negative exposure to transportation occurred during the fear period of development (8–10+ weeks), which is a period when exposure to aversive stimuli can cause permanent fear retention [11].

Although studies have explored indicators of puppies’ physical health in relation to transportation [12,13], no studies have examined its effects on comprehensive assessments of puppy welfare. Puppies from commercial breeding kennels (CBKs) provide a unique opportunity to do so, as large numbers are typically transported by road to a distributor at approximately 8 weeks old. This marks the beginning of their journey to a pet store or other destination to be sold. Transportation may act as a general stressor that puppies from CBKs inevitably undergo, which may increase generalized fear and translate into future behavioral problems. Previous studies based on owner-reports cited higher levels of behavioral issues in puppies from pet stores, believed to originate from large-scale breeding establishments [13]. However, since the provenance of these subjects was not traceable, causality could not be demonstrated, and the possible effects of travel stress on adult behavior should not be ruled out. As a large proportion of pet dogs in the U.S. originate from breeders and pet stores [14], and anecdotal reports indicate an increasing proportion are being sold online, optimizing transport conditions may safeguard the puppies’ welfare, as well as prevent the development of travel-related and other behavioral issues in adult pet dogs.

While previous studies have examined the behavior of adult dogs during road transportation, no study has compared their behavior in response to a mild stressor before and after transportation to gauge their affective state. Affective state is a major component of animal welfare and can be measured by examining an animal’s behavioral and physiological response to a mild stressor [7,15]. Previous studies in approximately 8-week-old puppies showed that brief periods of isolation were successful in eliciting a range of responses indicative of stress, such as vocalizations and the duration of locomotor and exploratory activity [16,17,18,19,20]. In CBKs specifically, response to stranger approach has proven to elicit a range of mild stress responses in adult dogs [21,22,23,24] and, more recently, in 8-week-old puppies [25].

Many studies have explored the effects of transportation on physiological parameters in adult dogs. Cortisol, which is a product of the hypothalamic-pituitary-adrenal (HPA) axis is commonly used to measure an individual’s physiological arousal [26], which may denote distress [27,28]. Studies have found an increase in adult dogs’ salivary [29,30,31], blood (i.e., plasma or serum) [30,32,33,34], and fecal [35] cortisol concentrations after road transportation, indicating that road transportation elicited significant arousal [26]. Stressful environmental and management conditions can also alter an animal’s immune function in a manner dependent on HPA axis and autonomic nervous system activation, as well as other physiological and behavioral changes [36]. For instance, multiple studies showed that adult dogs’ neutrophil: lymphocyte (N/L) ratios and white blood cell counts significantly increased after road transportation [29,30,31,37]. Conversely, one study found no significant difference in adult dogs’ serum cortisol concentrations and decreases in N/L ratios between measures immediately after road transport and 24 h later [38]. This finding likely differs from the previously mentioned study due to the lack of a baseline (i.e., before transportation) measurement. Further, the absence of a significant decrease in cortisol between the two time points supports the idea that dogs’ physiologic stress responses may take days to return to baseline after transportation in some populations [34,35].

Short-term methods of collection for physiological metrics such as saliva, serum, and plasma are difficult to gather in CBKs, as puppies from some kennels are not familiar with routine handling, and the handling related to these procedures can itself induce distress [39]. Further, longer-term collection metrics such as fecal sampling are less invasive than saliva or blood sampling. Because of this and the fact that dogs’ physiological stress responses to transportation are likely still present after multiple days [34,35], longer-term metrics of stress may be more appropriate to assess the effects of transportation on welfare. Farca et al. [35] were able to capture changes in adult dogs’ HPA axis activation in response to transportation using fecal glucocorticoid metabolites (FGM), which represent cortisol concentrations in dogs over approximately 24 h [40]. Fecal secretory immunoglobulin A (sIgA), which is the primary antibody of the mucosal immune system in most mammals, can be another viable measure in providing data over a longer-term scale [41,42]. Fecal sIgA concentration can change based on the intensity and duration of the stressor experienced, but it is generally upregulated in response to acute stress and downregulated in response to chronic stress [43]. To our knowledge, no study related to transportation in dogs has incorporated longer-term measures of immune response.

Therefore, the objective of the current study was to assess changes in behavioral, physical, and physiological indicators of puppy welfare after transportation from their kennels of origin to a distributor. We hypothesized that metrics would be significantly different post-transportation as compared to pre-transportation. To our knowledge, this is the first study to explore the effects of transportation on puppy welfare.

## 2. Materials and Methods

### 2.1. Ethics Statement

The Purdue University Institutional Animal Care and Use Committee approved all experimental procedures and sample sizes. Breeders and distributors volunteered for the study and were required to sign a consent form prior to commencement of experimental procedures. They were permitted to withdraw from the study at any point, for any reason. If, at any point, puppies exhibited signs of extreme distress or physical illness, they were removed from testing.

### 2.2. Subjects

Subjects (*n* = 383) were approximately 8-week-old puppies (litter metrics: mean = 57.5 days old, sd = 2.36, range = 54–64 days old; 192 males and 191 females) from 88 litters, comprised of 31 breeds or designer crossbreeds, from 12 CBKs in the U.S. Midwest (Section S1, Table S1). Data were collected from a minimum of six to a maximum of nine litters per facility to ensure sufficient representation of within kennel variations (e.g., breed, maternal factors). Further, multiple kennels were included to capture variations in management and environmental factors between facilities. The number of puppies enrolled per facility was intended to be larger than a similar study in adult dogs [35], to ensure significant differences could be detected at *p* < 0.05. The sample size accounted for data loss post-transport due to puppies not being transported because they were retained as breeding stock, sold to different brokers, or did not meet the minimum required weight (hence transported at an older age), or due to logistic impediments such as inclement weather, etc. All puppies were group-housed in pens of varying sizes (all exceeding USDA requirements) and flooring types (e.g., tenderfoot, tile). All puppies had continuous access to water.

### 2.3. Experimental Procedures

Data were collected from June 2019 to December 2021. Before entry into facilities, experimenters placed disposable boot covers (Innovative Haus, Premium Thick Waterproof Disposable Shoe Covers, Shelton, CT, USA) over their shoes and put on nitrile gloves (KIMTECH^TM^, Roswell, GA, USA), which were changed between testing of different litters. Puppies were tested at their kennels of origin, and after road transportation to a distributor (litter metrics: mean = 2.73 days in between testing days, sd = 0.45, range = 2–3 days). Puppies were transported to one of three distributors in the U.S. Midwest and were housed there for approximately 48 h before testing began. This period was allotted to allow puppies time to habituate to their new surroundings. Road transportation was free from experimental manipulation and was conducted in accordance with normal practices for each distributor. Travel time from kennels of origin to the distributor varied and was not always traceable. At both the kennel of origin and distributor locations, puppies were subjected to a one-minute isolation test and a version of the Field Instantaneous Dog Observation Tool (FIDO+) [21,22,23,24], which is a three-step stranger approach test and visual physical health assessment adapted for puppies. Three equally trained female experimenters conducted all behavioral tests, and in most cases, each puppy was tested by the same experimenter at both locations. Throughout the FIDO+, an additional research assistant was standing off to the side, recording the scores verbally dictated by the experimenter into a spreadsheet (Microsoft Excel, Redmond, WA, USA) on an iPad (Apple Inc., Cupertino, CA, USA). Furthermore, fecal samples were collected from puppies for analyses of FGM, fecal sIgA, and presence of intestinal parasites.

#### 2.3.1. Isolation Test

At both locations, puppies were first subjected to an isolation test. Litters were tested one after another, and puppies within litters were selected for testing in no particular order. The isolation pen was disinfected with Rescue^TM^ (Virox Technologies Inc., Oakville, ON, Canada) between litters, or anytime a puppy eliminated in the pen. Each puppy was removed individually from their pen by a research assistant or caretaker. They were placed in the middle of an exercise pen with six 30-inch-tall black wire panels (Precision Pet Products, Arlington, TX, USA), configured into a hexagon on top of six black rubber mats (Rubber-Cal Armor-Lock 3/8 in. × 20 in. × 20 in. Black Interlocking Rubber Tiles). A tripod with a portable camera (Sony Handycam HDR-CX405, Tokyo, Japan) was placed behind one of the panels, outside of the pen, to record the puppies’ behaviors for later scoring. Immediately after the puppy was placed in the pen, the experimenter stepped a few paces away from the pen and started a timer for one-minute. Throughout the one-minute test, experimenters, research assistants, and caretakers were quiet and did not engage or make direct eye contact with the puppy being tested. Puppies’ behaviors during the test were continuously scored from video using the behavioral coding software BORIS [44] and the ethogram outlined in Table 1. Videos were coded by three independent coders after inter-rater reliability (IRR) analyses. Coders were assigned different puppies pre- and post-transportation to avoid bias.

#### 2.3.2. FIDO+

Immediately after the one-minute isolation test, while the puppy was still in the isolation pen, the experimenter conducted the FIDO+. The procedure and scoring were as follows.

Approach: The experimenter approached the pen and crouched down on one knee while maintaining a sideways orientation and averted gaze. The experimenter tossed a treat to the puppy (0.5 cm pieces of Canine Carry Outs—Beef Flavor or Pup-Peroni—Original- Beef Flavor, Big Heart Pet Inc., Walnut Creek, CA, USA) through the bars of the pen, and recorded whether or not they ate it.

Open: The experimenter opened the pen door while crouched on one knee and maintaining a sideways orientation with averted gaze. The experimenter reached one hand toward the puppy to offer them a treat and recorded whether or not they ate it.

Reach: The experimenter, while still maintaining the same orientation and gaze, simultaneously reached toward the puppy with one hand to offer them a treat, and the other hand to touch them on the shoulder, back, or chest. The puppy was only touched if they were in arm’s reach and interacting with and/or oriented toward the experimenter. The experimenter recorded whether or not the puppy ate the treat.

Throughout the test, the experimenter often made soft ‘kissing’ noises or tapped on the side of the pen to produce mild auditory stimuli. As puppies may not have had adult level visual acuity but had a developed startle response at this age [10], this was done to orient the puppy toward the experimenter, to ensure the puppy was aware of their presence.

Puppies’ behavioral responses were scored from their videos using the ethogram outlined in Table 2. For each step, every puppy was given a score for the orientation, response, and posture categories, and if applicable, they were also given scores for the modifier and additional categories (Table 2). All videos were scored by one coder (AR) to eliminate the possibility of variability in scoring between different coders. The FIDO+ tool has previously been evaluated for reliability [23], therefore, reliability was not a main goal of the current study.

The visual physical health assessment was conducted immediately after the behavioral portion of the FIDO+. It was adapted for puppies from Bauer et al. [22], and scored as follows: body condition score (BCS) on a scale of 1–3 (1 = thin, 2 = normal, 3 = obese), cleanliness based on percentage of body covered in debris (0%, 1–25%, 26–50%, 51–75%, 76–100%), tear staining based on severity (none, mild, moderate, severe), and presence or absence of nasal discharge, ocular discharge, sneezing, coughing, symptoms of upper respiratory infection (URI), diarrhea, poor coat condition, wounds, pyoderma (skin infection), and evidence of swimmer puppy syndrome (puppy ‘paddling’, unable to walk).

#### 2.3.3. FGM, Fecal sIgA, and Presence of Intestinal Parasites

Experimenters collected fecal samples from each litter (i.e., any feces excreted in a pen where the litter was housed) and one ‘focal’ puppy from each litter, which was randomly selected (random number generator, Google) by experimenters before the 8-week visit. This was necessary due to the labor-intensive nature of this type of data collection, which would have been difficult to carry out on all the puppies. Breeders and distributor staff were instructed to keep litters housed together. To obtain the focal puppy sample, they were instructed to feed each focal puppy a pea-sized amount of blue food coloring (Wilton, Royal Blue Icing Color, Woodridge, IL, USA) mixed with baby food (Turkey, Ham, or Chicken and Gravy, Gerber, Nestle, Florham Park, NJ, USA), 10–12 h before the experimenter’s visit was scheduled. Food coloring, baby food, and labelled plastic baggies for fecal samples were provided by experimenters before each visit. On the morning of the experimenters’ visit, breeders and distributor staff were instructed to collect spontaneously voided litter and focal puppy feces in each pen, place them in the corresponding baggies, and store them in a cool location. If this was not feasible, experimenters collected feces during their visit. Variation in time of fecal collection would not have affected results, as fecal metrics are representative of longer-term time frames that are unlikely to capture diurnal variations [39,42,53]. Once experimenters collected feces, they were split into two equal parts and placed in separate labelled baggies for FGM/fecal sIgA and intestinal parasite analyses. Feces were analyzed for intestinal parasites to aid in collecting a comprehensive overview of puppies’ general health, and not necessarily intended to be attributed to transportation. Transportation times and the total study duration were relatively short, leading to the inability to draw such conclusions. However, as this association has not been previously explored in puppies, the information was collected to form the basis for doing so in the future. While travelling to and from facilities, fecal samples were stored in a Styrofoam cooler with ice packs, and, if available on longer trips, in a refrigerator (approximately 1–3 °C). Storage while travelling ranged from 4 to 84 h. Once travel was completed, feces for FGM and sIgA analyses were stored in a freezer (−20 °C) until they were shipped to the laboratory for analyses (see below). Feces for parasite analyses were stored in a refrigerator (4 °C) for no longer than 1 week and analyzed at Purdue.

##### FGM and Fecal sIgA Sample Processing

Fecal samples were shipped on dry ice to Omaha’s Henry Doorly Zoo and Aquarium Endocrinology Lab in four batches. Samples were thawed and processed separately for cortisol and sIgA. Processing for cortisol involved drying >1 g of feces in a 60 °C oven for 48–72 h, to account for variation in fecal water content. Dried samples were pulverized into a fine power and 0.5 g was extracted by mechanically shaking overnight in 5 mL of 80% v:v methanol. Samples were centrifuged at 3000× *g* for 15 min and the supernatant stored at −20 °C until analysis. Processing for sIgA involved weighing 0.5 g of wet feces, adding 1.5 mL of PBS (524650, EMD Millipore, Burlington, MA, USA) containing protease inhibitor (cOmplete mini [1 tablet/10 mL PBS], Roche, Basel, Switzerland), vortexing for 30 s, and incubating for 60 min at RT, prior to a double centrifugation step (3000× *g* 15 min). The supernatant was collected and frozen until sIgA analysis within 1 month.

##### Cortisol Enzyme Immunoassay

Fecal cortisol concentrations were quantified by enzyme immunoassay (EIA) using an anti-cortisol antiserum (R4866) and cortisol-horseradish perioxidase (HRP) ligand (C. Munro, University of California, Davis, CA, USA). The polyclonal antiserum raised in rabbits was directed against cortisol-3-carboxymethyloxime (CMO), linked to bovine serum albumin and was shown to cross react with cortisol (100%), prednisolone (9.9%), prednisone (6.3%), cortisone (5%) and <1% with androstenedione, androsterone, corticosterone, desoxycorticosterone, 11-desoxycortisol, 21-desoxycortisone and testosterone [54]. The EIA was performed according to the methods established by Munro & Lasley [54]. Absorbance was measured at 405 nm (SpectraMax ABS, Molecular Devices, San Jose, CA, USA) and data extrapolated via 4-parameter curve fit using Softmax Pro 7.1 (Molecular Devices, San Jose, CA, USA). Results of each sample were expressed as ng of cortisol per gram of dry mass feces (dmf).

Inter-assay coefficients of variation for the cortisol assay were 3.9% and 5.4% for internal controls at 54% (20 pg/well) and 33% (70 pg/well) binding, respectively, for the first batch, 5.5% and 7.7% for internal controls at 53% (25 pg/well) and 32% (75 pg/well), respectively; for the second batch, 3.42% and 4.48% for internal controls at 62.24% (20 pg/well) and 34.47% (70 pg/well) binding, respectively, for the third batch. An inter-assay coefficient of variation was not calculated for the fourth batch as there was only one assay.

##### sIgA Enzyme-Linked Immunosorbant Assay

Fecal sIgA concentrations were quantified by a commercial enzyme-linked immunosorbant assay (ELISA) (E40-104-26, E101, Bethyl Laboratories, Montgomery, TX, USA) as follows: 96-well plates were coated for 1 h at RT with a 1:100 dilution of affinity purified goat anti-canine IgA antibody in 100 μL of coating buffer (0.05 M Carbonate-Bicarbonate, pH 9.6). Following coating, plates were washed five times (50 mM Tris, 0.14 M NaCL, 0.05% Tween 20, pH 8.0) before adding 200 μL/well of blocking solution (50 mM Tris, 0.14 M NaCL, 1% BSA, pH 8.0) and storing overnight at 4 °C. The next day, plates were washed five times before adding 100 μL/well of duplicate standards (15.6–1000 ng/mL) or samples and incubating 1 h at RT on a light-protected plate shaker (600 rpm). Plates were then washed an additional five times prior to adding 100 μL/well of a 1:75,000 dilution of goat anti-canine IgA:HRP in ELISA buffer (50 mM Tris, 0.14 M NaCL, 1% BSA, 0.05% Tween 20) and incubating for 1 h at RT on a light-protected plate shaker (600 rpm). A final wash step was performed before adding 100 μL/well of 3,3′,5,5′-tetramethylbenzidine (TMB) substrate solution and incubating the plate for 15 min at RT on a light-protected plate shaker (600 rpm). The reaction was stopped with 100 μL/well of 0.18 M H2S04 and absorbance was measured at 450 nm (SpectraMax ABS, Molecular Devices, San Jose, CA, USA) with data extrapolated via 4-parameter curve fit using Soft-Max Pro 7.1 (Molecular Devices, San Jose, CA, USA). Results for each sample were expressed as mg of sIgA per gram (wet weight) of feces.

##### Presence of Intestinal Parasites

The canine fecal samples were stored at 4 °C until a centrifugal fecal floatation assay using Sheather’s sugar solution (SPG 1.25) could be performed to detect the presence of parasite ova and/or cysts. Approximately one gram of the feces was placed in a paper cup containing 15–20 mL of the flotation solution and mixed thoroughly. This mixture was strained through a cheesecloth, and the solution was collected into a 15 mL centrifuge tube until it formed a convex meniscus. A cover glass was placed on this fluid meniscus and gently pressed-down such that it was securely seated on the rim of the centrifuge tube. This centrifuge tube with the cover glass on it was spun at 1500 rpm for 7 min in a swinging head centrifuge. After the centrifugation was complete, the cover glass was gently removed straight up, placed on a microscopic slide, and examined under a 10× objective lens of a compound microscope to detect parasite eggs/oocysts/cysts [55].

### 2.4. Statistical Analyses

Statistical analyses were performed using R version 4.1.3 (R Core Team, Vienna, Austria) and SAS 9.4 (SAS Institute Inc., Cary, NC, USA). Prior to analyses, data were tested for normality using the Shapiro–Wilk test, checking skewness and kurtosis, and visual inspection of the normal plot. The criterion for statistical significance was established at *p* < 0.05 and statistical trends were set at 0.05 < *p* < 0.10.

#### 2.4.1. Isolation Test

The individual puppy was considered the experimental unit. Body trembling, frozen, grooming, and play behaviors were not included in the final analysis as there were limited observations (i.e., zero inflated). The frequencies of elimination, body shaking, paw lifting, and lip licking were summed to create a ‘sum of stress-related behaviors’ variable, as there was low variation between individuals for each variable on its own. All four of these behaviors are indicative of acute stress in dogs [29,45,56]. To explore the effect of transportation on puppies’ behaviors, different cross-classified multilevel models were used, depending on the nature of the variables. The duration of exploration and locomotion behaviors were analyzed using the MIXED procedure of SAS, the duration of escape attempt and stationary behaviors were analyzed using the GLIMMIX procedure of SAS with gamma distribution and log link function, whereas the frequency of escape attempt and sum of stress-related behaviors were analyzed using the GLIMMIX procedure of SAS with Poisson distribution and log link function. The previously stated behaviors were used as dependent variables. Transportation (i.e., pre- and post-) and sex were included as fixed effects, whereas facility and puppy ID within Dam ID were included as random effects to account for any facility or litter effects. Behavioral variation was observed among facilities regardless of the time point (i.e., pre- and post-transportation), thus descriptive statistics in the MEANS procedure of SAS were used to investigate differences among facilities within and between time points and were presented as means ± SD. Results of fixed effects are presented as least squares means ± SE while results of random effects are presented as estimates ± SE. A Tukey–Kramer adjustment was used to account for multiple post hoc comparisons. Spearman and Pearson’s correlations were also used to investigate consistencies in puppies’ behaviors across time points, and results are presented as a correlation coefficient (Rho).

#### 2.4.2. FIDO+ (Behavior)

The FIDO+ was converted into a numerical score as follows: Orientation (Yes = 1, No = 0), Approach (Approach = 2, Ambivalent Approach = 1, No Approach = 0), Modifier (Affiliative-Outgoing = 2, Affiliative = 1, Undisturbed = 0, Stationary = 0, Avoid = −1), Posture (Normal = 1, Low = 0), Treat (Yes = 1, No = 0), Touch (Yes = 1, No = 0). These scores were summed to create an overall score (FIDO+ sum, maximum points = 22).

As before, the individual puppy was considered the experimental unit. To explore the effects of transportation on the FIDO+ sum, a cross-classified multilevel model in the MIXED procedure of SAS was used. FIDO+ sum score was used as the dependent variable. Transportation (i.e., pre- and post-) and sex were included as fixed effects, whereas facility and puppy ID within Dam ID were included as random effects. FIDO+ sum score variation was observed among facilities regardless of time point (i.e., pre- and post-transportation), thus descriptive statistics in the MEANS procedure of SAS were used to investigate differences among facilities within and between time points and were presented as means ± SD. Results of fixed effects are presented as least squares means ± SE, while results of random effects are presented as estimates ± SE. A Tukey–Kramer adjustment was used to account for multiple post hoc comparisons. A Pearson’s correlation was used to investigate a possible association of the FIDO+ sum score between time points, and results are presented as a correlation coefficient (Rho).

#### 2.4.3. IRR

IRR was assessed between observers for both the isolation test and FIDO+ scoring variables. Analyses and results (Tables S2 and S3) are detailed in Section S2 of the Supplementary Materials.

#### 2.4.4. Physiological Data

Focal puppy and litter were both considered experimental units and data were analyzed separately. To explore the effect of transportation on FGM (ng/g) and sIgA (mg/g) concentrations, cross-classified multilevel models in the GLIMMIX procedure of SAS with gamma distribution and log link function were used. Before statistical analysis, data editing was performed to remove outliers that did not fall within the range of mean ± 3 SD. FGM and sIgA were used as dependent variables. Transportation (i.e., pre- and post-) and sex were included as fixed effects, whereas facility and Dam ID were included as random effects. FGM and sIgA variation were observed among facilities regardless of time point (i.e., pre- and post-transportation), thus descriptive statistics in the MEANS procedure of SAS were used to investigate differences among facilities within and between time points and were presented as means ± SD. Results of fixed effects are presented as least squares means ± SE, while results of random effects are presented as estimates ± SE. A Tukey–Kramer adjustment was used to account for multiple post hoc comparisons. Spearman’s correlations were used to investigate possible associations of FGM and sIgA concentrations within and between time points at both focal puppy and litter levels, and results are presented as a correlation coefficient (Rho).

#### 2.4.5. Physical Health and Parasites

Descriptive statistics were used to investigate the physical health status of the puppies. Cleanliness scores were excluded from the final analyses, as it was standard practice for distributors to bathe and groom puppies upon arrival, resulting in the inability to attribute changes in cleanliness to transportation itself. The MEANS and FREQ procedures of SAS were used to calculate the number and percentage of puppies affected by each health condition and type of parasite pre- and post- transportation.

## 3. Results

Four puppies were excluded from the final analyses as they were not raised by their biological dams, or they were raised in a household environment rather than a kennel. Further, four litter-level fecal samples were excluded from the final analyses as they may have been contaminated by cross-fostered puppies. Therefore, the final analyses included 379 puppies (Section S1, Table S1).

### 3.1. Isolation Test

Overall, transportation had a significant effect on the durations and frequencies of puppies’ behaviors during the isolation test (*p* < 0.01; Table 3). Specifically, puppies performed longer durations of escape attempt behaviors and locomotion post-transportation, as opposed to pre-transportation, whereas shorter durations of exploration and stationary behaviors were observed post-transportation compared to pre-transportation (*p* < 0.01; Table 3). No significant effects of sex were reported for any of the studied behaviors (*p* > 0.05). A significant variation was reported among facilities for duration of locomotion (*p* = 0.03) regardless of time point, whereas duration of escape attempt and stationary behaviors tended to differ among facilities (Table 3). Similar variation was observed at the litter level (Dam ID) for duration of exploration and locomotion behaviors (*p* < 0.05; Table 3). Descriptive statistics showed overall variability in duration of exploration, locomotion, escape attempts, and stationary behaviors among facilities, both within and between time points. These data are presented as Supplementary Material (Section S3, Figure S1).

All observed behaviors were positively correlated between time points (*p* < 0.01) even though the strength was low to moderate: duration of locomotion (Rho = 0.19), exploration (Rho = 0.24), stationary (Rho = 0.19), and escape attempt behaviors (Rho = 0.23).

Frequencies of escape attempt behaviors were higher post-transportation (0.49 ± 0.09), as compared to pre-transportation (0.23 ± 0.04; *p* < 0.001), while no significant results were reported for frequencies of stress-related behaviors (0.96 ± 0.12 vs. 1.00 ± 0.13, pre- and post-transportation, respectively; *p* = 0.608). No significant effect of sex was reported for either of the behaviors (*p* > 0.05). A significant variation was reported among facilities for frequencies of stress-related behaviors (0.145 ± 0.075; *p* = 0.028), regardless of time point, whereas no significant results were found for frequencies of escape attempt behaviors (0.133 ± 0.115; *p* = 0.123). Similar variation was observed at the litter level (Dam ID) for frequencies of stress-related (0.503 ± 0.080; *p* < 0.001) and escape attempt behaviors (2.142 ± 0.353; *p* < 0.001).

Frequencies of stress-related (Rho = 0.29) and escape attempt behaviors (Rho = 0.25) were positively correlated between time points (*p* < 0.001).

### 3.2. FIDO+ (Behavior)

**Figure 1:**
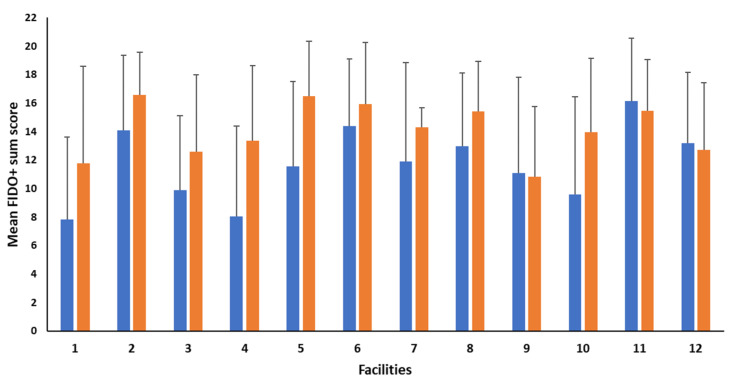
Descriptive statistics of the FIDO+ sum score variation among facilities pre- and post-transportation. Differences in FIDO+ sum score among facilities within and between time points (i.e., blue bars = pre-transportation: puppies of 8-week of age assessed at the CBK; orange bars = post-transportation: puppies assessed 48 h after arrival to the distributor).

Transportation had a significant effect on FIDO+ sum scores (*p* < 0.001), with puppies scoring higher (i.e., more affiliative) post-transportation (14.22 ± 0.64) when compared to pre-transportation (11.61 ± 0.62). Sex did not affect this metric (12.78 ± 0.65 vs. 13.05 ± 0.66, males and females, respectively; *p* = 0.592). A significant variation was also reported among facilities (3.591 ± 1.828; *p* = 0.025) and Dam ID (9.883 ± 1.995; *p* < 0.001), regardless of time point. Descriptive statistics of the FIDO+ score variation among facilities, within and between time points, are presented in Figure 1.

A positive correlation was reported between pre- and post-transportation for the FIDO+ sum scores (Rho = 0.40; *p* < 0.001).

### 3.3. Physiological Data

**Figure 2:**
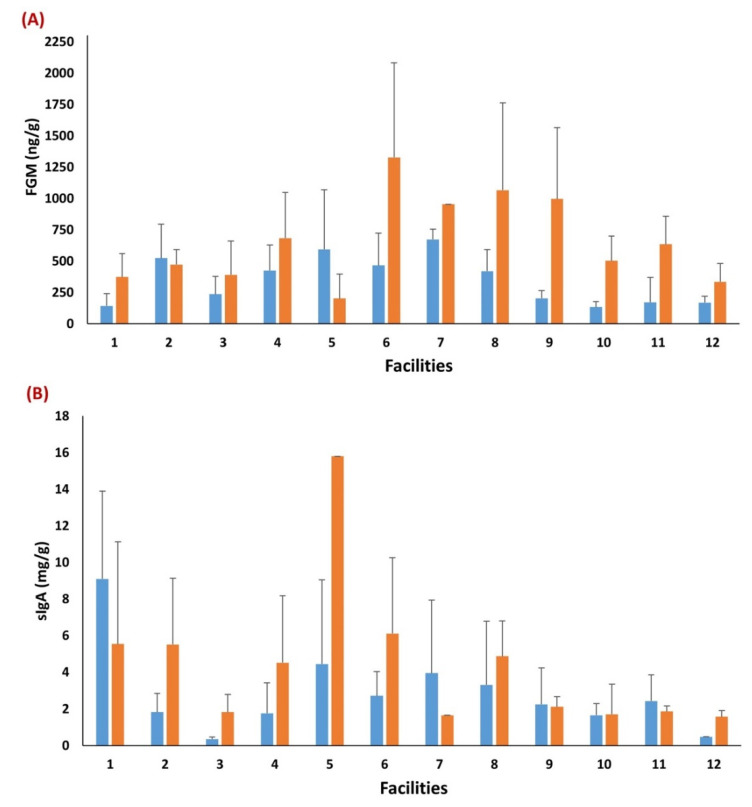
Descriptive statistics of the variation in focal puppies’ FGM (ng/g) and sIgA (mg/g) concentrations among facilities, pre- and post-transportation. Differences of FGM (**A**) and sIgA (**B**) concentrations among facilities within and between time points (i.e., blue bars = pre-transportation: puppies of 8-week of age assessed at the CBK; orange bars = post-transportation: puppies assessed 48 h after arrival to the distributor).

Transportation had a significant effect on focal puppies’ FGM and sIgA concentrations (*p* < 0.01; Table 4), with puppies having higher FGM (665.43 ± 97.73) and sIgA (3.55 ± 0.73) concentrations post-transportation when compared to pre-transportation (FGM = 306.07 ± 42.69; sIgA = 2.31 ± 0.47). There were statistically significant differences among facilities for both FGM (*p* = 0.032) and sIgA (*p* = 0.029), regardless of time point (Table 4), while Dam ID had no significant effect on either metric. Descriptive statistics are presented in Figure 2, reporting an overall variation in FGM and sIgA among facilities within and between time points. A positive association was reported between pre- and post-transportation FGM concentrations (Rho = 0.42; *p* = 0.004), while there was no association of sIgA concentrations (Rho = 0.19; *p* = 0.262) between time points.

At the litter level, transportation had a significant effect on FGM (*p* < 0.001; Table 5), with litters having, on average, higher concentrations post-transportation (750.57 ± 59.82 ng/g) compared to pre-transportation (330.25 ± 55.57 ng/g). No significant effect was found for sIgA between time points. Both FGM (*p* = 0.055) and sIgA (*p* = 0.056) tended to differ among facilities, regardless of time point (Table 5), whereas Dam ID had no significant effect on FGM only. A positive correlation was reported between pre- and post-transportation for FGM (Rho = 0.51; *p* < 0.001) and sIgA concentrations (Rho = 0.42; *p* = 0.002).

Positive correlations were identified between focal puppy and litter FGM and sIgA both pre- (FGM- Rho = 0.53, *p* < 0.0001; sIgA- Rho = 0.59, *p* < 0.0001) and post-transportation (FGM- Rho = 0.61, *p* < 0.0001; sIgA- Rho = 0.57, *p* = 0.0002).

### 3.4. Physical Health and Parasites

Descriptive data showed that puppies were physically healthy both pre- and post-transportation. The number and percentage of puppies observed pre- and post-transportation with each health condition are shown in Table 6 with tear staining being the most common, followed by ocular discharge (8.8% vs. 17.0%). Overall, no swimmer syndrome was reported during either time point, while less than 1% of the puppies had other, more serious health concerns such as wounds, diarrhea, URI, coughing, or nasal discharge.

The majority of the puppies assessed (48 vs. 40 out of 88 puppies pre- and post- transportation, respectively) did not have intestinal parasites at either time point (70.8% vs. 80%). *Giardia* sp. was the most frequently observed parasite both pre- and post-transportation (16.7% vs. 12.5%, respectively), followed by *Cystoisospora ohioensis complex* (12.5% vs. 7.5%). *Cystoisospora canis* (4.72%, *n* = 2 puppies) and *Cryptosporidium* sp. (2%, *n* = 1 puppy) were only reported in puppies assessed pre-transportation. Only three out of 88 puppies were affected by more than one type of parasite. Variation of the number of puppies affected by parasites was observed among facilities (mean = 1.8, median = 1), with eight out of 12 facilities having at least one puppy affected by parasites, regardless of time point. Of those facilities, only two had puppies affected by more than one type of parasite.

## 4. Discussion

This is the first study to explore the effects of transportation on puppy welfare. Our initial hypothesis was confirmed, as we found significant changes in behavioral, physiological, and physical metrics of puppy welfare after transportation from CBKs to a distributor. Findings indicate good biological health for puppies at both time points. However, changes in behavioral and physiological metrics suggest increased puppy distress following ground transportation to a distributor.

### 4.1. Isolation Test and FIDO+

During the isolation test, puppies exhibited significantly higher escape attempt frequencies and durations post-transportation as compared to pre-transportation, a result which highlights their increased motivation to leave the stressful scenario and may be indicative of distress [57]. Puppies also displayed significantly less exploration and stationary behavior, and more locomotion post-transportation as compared to pre-transportation. Literature suggests reduced exploration may be indicative of fear [58]. In a study by Guardini et al. [46], puppies who exhibited more exploration during an isolation test also showed fewer signs of distress such as a longer latency to first yelp, reduced destructiveness and non-exploratory locomotion, and fewer escape attempts and vocalizations. Further, Elliot and Scott [56] identified more active behavior, elimination, and vocalizations when puppies were isolated in an unfamiliar pen as compared to their home pen, which they concluded were signs of increased distress. Therefore, the changes in behaviors from pre- to post-transportation exhibited by puppies in the current study are likely indicative of increased distress. An increase in the frequency of stress-related behaviors (i.e., elimination, body shake, paw lift, lip lick) was expected, but the lack of significant change was not surprising, given the low frequencies of these behaviors. This may be due to isolation acting as a mild stressor, which in turn elicits less intense stress responses. The low frequencies may have also led to difficulties in detecting these behaviors. Additionally, the lack of significant difference may be explained by the reduced capability of puppies to perform stress-related behaviors with the same precision as adults, due to their ongoing development of fear-related behaviors and mobility during this time [59]. This may have led to their lack of performance of such behaviors or the inability of coders to detect them.

In contrast to patterns observed during the isolation test, results from the FIDO+ indicated puppies showed more affiliative behavior toward an unfamiliar person post-transportation as opposed to pre-transportation. Therefore, as distress during isolation increased, so did puppies’ positive responses to the social stimulation of an unfamiliar person approaching immediately after the test ended. Guardini et al. [60] found similar results in approximately 8-week-old puppies, in that increased distress during separation was associated with increased engagement with an unfamiliar person when an attachment figure was not present. Further, Pettijohn et al. [61] found that active human contact (as compared to food, inanimate objects, and canine contact) was most effective in alleviating puppy distress following isolation. Therefore, it is plausible that the more distress puppies experienced, the more comfort they were likely to seek from human approach.

The significant positive correlations between pre- and post-transportation measures of all behaviors during the isolation test and FIDO+ sum scores indicate that puppies’ behaviors during the isolation test and their reactions to an unfamiliar person afterwards were consistent before and after transportation. Findings also indicated facility and/or litter effects were responsible for variation in behaviors during the isolation and stranger approach tests. Management and environmental factors varied by facility, and variations in them have recently been found to be associated with adult dogs’ behaviors both in CBKs and when rehomed [62]. Therefore, it is plausible they were also responsible for variations in puppies’ responses to stressors in the current study. For example, it is likely that puppies that were exposed to novel environments, similar stimuli to that experienced during the isolation test, and unfamiliar people when at their kennels of origin, may have shown fewer behaviors indicative of distress when subjected to isolation, and more affiliative behavior toward the experimenter. In addition, caretaker-puppy interactions likely played a role, as some facilities showed little change in FIDO+ score from pre- to post-transportation (Figure 1). This indicates that although distress during isolation may have increased, puppies from some facilities were not more comforted by the unfamiliar person’s presence, which may be due to the quantity and quality of caretaker interactions they experienced early in life. In addition, multiple maternal factors such as dam exposure to prenatal stress, genetics, and maternal care may have caused the significant effect of the dam on puppy behavior. For example, multiple studies have identified maternal care is related to 8-week-old puppies’ behaviors in an isolation or arena test, as well as their responses to an unfamiliar person [46,60]. Future studies are needed to further explore the effects of specific facility and dam-related factors on puppy behavior.

### 4.2. Physiological Data

In addition to behavioral changes, focal puppies and litters exhibited a significant increase in FGM and focal puppies exhibited a significant increase in sIgA after transportation. Although there was an increase in mean litter sIgA concentrations from pre- to post-transportation, the difference was not statistically significant. This may be due in part to samples being collected at the litter level, meaning the same individual’s sample might not have been represented in the data at both time points, which may have diminished the effect that was observed in focal puppies. Further, as the average magnitude of concentration change from pre-to post-transportation was larger in cortisol than sIgA, it may have been easier to detect a statistically significant difference in litter FGM as opposed to litter sIgA. However, it is important to note that there were strong positive correlations between collection methods of the same metric within time points and the data revealed the same pattern regardless of collection method. Therefore, collecting litter level data as opposed to data from individual focal puppies may be sufficient for future studies and the decision should be weighed against budget and time constraints.

Increases in FGM and sIgA concentrations after transport support previous studies in adult dogs, citing a significant increase in cortisol [29,30,31,32,33,34,35] and upregulation of the immune response after ground transportation [29,30,31,37]. Higher concentrations of FGM after transportation are indicative of increased HPA axis activation and arousal in puppies but cannot provide specific information as to whether they experienced eustress or distress [26]. Measures of immune response, specifically sIgA, have been suggested to help in elucidating the valence of a stressor [42], but the relationship remains unclear. Theoretically, increased sIgA concentration is indicative of acute stress, but this can be significantly influenced by the intensity of a stressor [43]. Adding to these contradictory results are studies identifying higher basal concentrations of sIgA associated with more confident and less anxious behavior in adult cats and dogs [63,64,65]. It is also important to note that IgA concentration varies by age [66,67,68], and therefore it is plausible its response to stress may, as well. To our knowledge, only one study has explored the relationship between sIgA and stress in puppies and found that 7-week-olds showed a non-statistically significant increase in salivary sIgA after acute exposure to novel stimuli [69]. Nonetheless, age should not have confounded the significant increase in sIgA we observed in the current study, as there was only an approximately 3-day age gap between the two testing days. Overall, it is important to interpret these physiological findings alongside behavioral findings, as there is no single metric that is indicative of animal welfare [70]. Interpreting multiple metrics together provides insight on puppies’ overall welfare states rather than specific aspects. Therefore, taken together with the behavioral results that indicate increased puppy distress, it is likely that changes in puppies’ physiologic states after transportation to a distributor are also indicative of increased distress.

Significant positive correlations between pre- and post-transportation focal puppy and litter FGM concentrations suggest that those puppies that were more aroused at their kennel of origin were also more aroused at the distributor. There was also a significant positive correlation between litter sIgA concentrations from pre-to post-transportation, but not focal puppy concentrations. As the change in litter concentrations was less precise than the change in focal puppy metrics, there is a chance this finding was due to error. Therefore, the positive correlation between litter sIgA from pre- to post-transportation should be interpreted cautiously. The lack of relationship in the focal puppies may indicate that multiple variables were affecting sIgA concentration, resulting in an inconsistent relationship between time points.

The significant effect of facility on focal puppy FGM and sIgA indicates differences in environmental and management practices between CBKs likely contributed to the variation in concentrations. The same effect was not significant for litter data, even though there was a trend in the same direction. In terms of cortisol, socialization practices may have played a role in these differences, as a previous study in adult dogs in CBKs found an increased number of socialization practices was associated with decreased hair cortisol concentrations [24]. In addition, the quantity and quality of caretaker interactions likely differed between CBKs and Dudley et al. [71] identified that positive human-dog interactions have been associated with a decrease in adult dogs’ plasma cortisol concentrations. There may be different environmental and management factors that also influence immune function, as Dudley et al. [71] found no effect of positive-caretaker interactions on immunologic parameters. Further, no significant correlation was found between FGM and fecal sIgA in 8-week-old puppies from CBKs, indicating the same factors that may influence cortisol concentration may not influence sIgA at this age [72]. It is also important to note that not only did facility influence overall FGM and sIgA concentrations, but also the descriptive results depicted in Figure 2 indicate the direction and magnitude of their change from pre- to post-transportation varied by facility. This suggests that management and environmental factors likely contributed to suppressing or exacerbating the amount of physiologic distress experienced after transportation to a distributor. Further research must be conducted to determine specific facility-related factors that affect puppies’ physiologic profiles and how those factors may minimize puppy distress after a significant stressor.

### 4.3. Physical Health and Parasites

Most puppies had no major physical health concerns before or after transport. This complements the plethora of previous literature identifying that adult dogs in CBKs are physically healthy [24,73,74]. It is plausible that the increase in the percentage of puppies with ocular discharge post-transportation may be related to the stressor of ground transportation to a distributor. In cats, ocular discharge is often an indicator of stress or disease and is positively related to length of stay in a shelter [75]. Future studies are needed to further explore this relationship in puppies.

The most parsimonious explanation for the slight increase in percentage of puppies with no intestinal parasites post-transportation is that puppies were treated with an antiparasitic agent upon arrival to the distributor. If this was true, the agent would likely not have affected fecal sIgA concentration, therefore still maintaining its ability to provide insight regarding puppies’ responses to stress [76]. However, it is important to note that experimenters could not confirm whether antiparasitic agents were administered. Therefore, changes observed may have been associated with the observed changes in immune function. As sIgA aids in eliminating pathogens [41], it is plausible that the increase in sIgA associated with the acute stressor of transportation to a distributor was related to the reduction in percentage of puppies with intestinal parasites. Evidence that sIgA is negatively associated with parasitic load in dogs supports this explanation [77]. However, these results should be interpreted with caution, as more research is needed to determine the relationship between parasitic load, measures of immune function, and stress. Unfortunately, because the use of antiparasitic agents at the distributor could not be ruled out, no conclusions can be drawn about the effect of ground transportation on the presence of intestinal parasites in puppies. Future studies may be able to study this association with longer transport times, especially those that include multiple stops for feeding and watering, study durations, and information regarding breeder and distributor antiparasitic practices. Nevertheless, in the current study, most puppies were free from intestinal parasites both pre- and post-transportation, further supporting that most puppies had no major physical health concerns that may have negatively impacted their welfare.

### 4.4. Limitations

The limitations of the current study include the inability to disentangle the effects of ground transportation and arrival in a novel environment on puppy welfare. As this is the first study on puppy transportation and welfare, it was important to identify if and how puppies’ welfare changed after transportation to a distributor, before exploring specific transportation-related factors that may affect their welfare. Another limitation is that fecal masses were split into two equal parts for intestinal parasite and FGM and sIgA analyses. Literature in some species suggests that IgA and cortisol are not evenly distributed throughout fecal masses [78,79,80]. Future research should pay more attention to homogenize the fecal material before analysis. Furthermore, as participation in this study was voluntary, we are unable to conclude that the results are representative of all U.S. CBKs. Finally, it is plausible that the familiarity of the experimenters and testing protocols due to repeated measures may have influenced puppies’ post-transportation behavioral responses. If this occurred, however, we would have expected puppies to exhibit fewer behaviors indicative of distress during the isolation test with increased familiarity (i.e., post-transportation), which was not the case. Although recent studies on adult dogs’ FIDO+ responses in CBKs found no effect of test repetition across three consecutive days [21], we cannot completely discount an effect of repeated measures on puppies’ FIDO+ responses.

### 4.5. Implications and Future Directions

Our findings may provide insight into factors that may contribute to the owner-reported increase in behavioral problems in puppies bought from U.S. pet stores [81]. Stress experienced early in life, especially in the fear period of development, can dramatically alter puppies’ later behaviors and abilities to cope with stressors [11]. As we saw evidence of increased puppy distress following transportation to a distributor, it is possible that the experience induced generalized fear and fear toward transportation-related stimuli in puppies, leading to later issues.

The current study provides a basis for future studies on puppy transportation in CBKs. Changes in physiological and behavioral indicators of puppy welfare suggest increased puppy distress following transportation to a distributor, making it important to develop science-based interventions to mitigate potential negative outcomes. As we found that puppies may have attempted to cope with stress by seeking human-contact post-transportation, studies should capitalize on the likely benefits of these positive human–animal interactions. Future studies should also identify the effects of specific kennel/distributor practices (e.g., socialization, puppy-caretaker interactions) and transportation-related factors (e.g., transportation time, environmental conditions) on puppy welfare. Furthermore, as increased puppy distress was evident 48 h after transportation to a distributor, future studies should identify the long-term implications of this stressor on puppy welfare indicators (i.e., how long metrics take to return to baseline). Doing so will help inform distributor practices and procedures so as to not compound the amount of distress puppies may be experiencing. Future studies on these topics will aid in improving the welfare of puppies from CBKs in the short-term and into adulthood.

## 5. Conclusions

Overall, the current study found evidence of good physical health in puppies both before and after transportation. However, we also found behavioral and physiological evidence of increased puppy distress following ground transportation to a distributor, even after 48 h from arrival. Puppies may have attempted to mitigate this distress by seeking more social contact after transportation when subjected to an acute stressor. Further, puppies’ behavioral and physiological responses varied by facility and/or litter. Therefore, environmental, management, and maternal factors warrant further investigation as tools to improve puppies’ abilities to cope with the stressor of ground transportation to a distributor. Future studies should also explore the effects of transportation times and conditions and their long-term effects on puppy welfare.

## Figures and Tables

**Table 1:** Ethogram used to score puppy behavior in the isolation test.

Behavior	Description	Frequency	Duration	Presence/Absence
Locomotion	Walking or running around without exploring the environment or playing [45]	×	×	
Stationary	The puppy is in a static posture (i.e., sitting, lying). There may or may not be visual orientation toward the environment. The puppy may change posture in place but does not show any displacement [46]		×	
Frozen	The puppy is completely still while in any posture or position [22]	×	×	
Exploration	Activity directed toward physical aspects of the environment that could include sniffing, or examination such as licking or pawing [45,47,48,49]	×	×	
Play Behavior	A display of a number of predatory type behaviors such as mouse jumping, biting but changing position frequently, and play bowing, all displayed in a non-aggressive manner [50]	×	×	
Body Shake	“The puppy shakes his/her body” [45,46]	×		
Yawning	“Mouth open wide for a period of a few seconds, then closes” [46,51]	×		
Grooming	Behaviors directed towards the puppy’s own body. Like scratching, licking and biting-self [45]	×	×	
Lip Licking	“Tongue extends upwards to cover nose, before retracting into mouth” [46,51]	×		
Body Trembling	The puppy’s whole body is shivering for a minimum of 3 s [45]	×	×	
Paw Lift	“A fore paw is lifted into a position of approximately 45°” [45,46]	×		
Elimination	Urination and/or defecation	×		
Vocalization	Puppy barks, growls, howls, whines or makes any other auditory signal with their mouth [49]			× *
Escape Attempt	All active behaviors resulting vigorous contact with the pen fence, including scratching the fence with the paws, jumping on the fence, pulling on the fence with the forelegs or the mouth (including chewing, biting, shaking) [46]	×	×	

× Behavior was measured with the respective sampling technique. * Vocalization was initially scored as present or absent. Due to space constraints in kennels, isolation pens were often times placed near adult dogs’ pens, making it difficult to determine the source of the vocalization and score frequency objectively by video.

**Table 2:** Ethogram used to score puppies’ behavioral responses to each step of the FIDO+.

Category	Response	Definition
Orientation (Mutually exclusive)	Orientation	The puppy acknowledges the experimenter (i.e., makes eye contact/is oriented toward) within 7 s. * If selected, continue to score the remaining categories
	No Orientation	The puppy does not acknowledge the experimenter’s presence within 7 s (or the duration of the step). * If selected, only choose a ‘Posture’ and ‘Additional’ (not ‘Approach’ or ‘Behavior’)
Response (Mutually exclusive)	Approach	The puppy moves toward the experimenter (i.e., takes steps toward them, or leans toward them if they cannot step any closer).
	Ambivalent approach	The puppy approaches and retreats or approaches but then stops before reaching the experimenter. [22]
	No approach	The puppy does not approach (i.e., does not move toward the experimenter). [22]
Modifier (Mutually exclusive, if applicable)	Affiliative behavior	Any behaviors exhibited by the puppy intended to facilitate the establishment or reinforcement of a social bond. Examples include approaching the experimenter while maintaining eye contact and/or making physical contact (e.g., licking, touch) with the experimenter. Select if applicable: ▯ Outgoing The puppy jumps up or ‘scrambles’ at the front of cage and/or attempts to cross/crosses the front barrier of cage and/or exhibits repeated physical contact with the experimenter (e.g., repeatedly licking, jumping on hands, etc.) and/or approaches the experimenter while exhibiting intense tail wagging.
	Undisturbed	The puppy is engaging in an active behavior (e.g., sniffing, eating, etc.) when the step begins, then acknowledges the experimenter’s presence and returns to the same behavior. [22]
	Avoid	The puppy avoids the experimenter (i.e., moves away from them, turns their head in the opposite direction). [22]
	Stationary	The puppy is in a static posture (i.e., sitting, lying). There may or may not be visual orientation toward the environment. The dog may change posture in place but does not show any displacement. [46]
**Posture** (Mutually exclusive)	Normal	“Normal posture under neutral conditions” for specific breed and age. [52]
	Low	“Back rounded and/or legs bent…, head lowered.” [52]
**Additional** (If applicable)	Fight/Aggression	The puppy exhibits aggression (e.g., lunging, growling, teeth baring, etc.). [22]
	Bark	Select one: ▯ Negative affect Barking associated with avoidance, aggression, frustration, etc. ▯ Positive affect Barking associated with play, greeting, excitement, etc.
	Stereotypic behavior	The puppy performs a pattern of behavior repeatedly (e.g., pacing, circling, etc.). [22]

**Table 3:** Effect of transportation, sex, facility, and Dam ID on the duration (sec.) of exploration, locomotion, escape attempt, and stationary behaviors exhibited by puppies during the isolation test (*n* = 367).

**Fixed Effects**		**Exploration (s)**			**Locomotion (s)**			**Escape attempt (s)**			**Stationary (s)**		
		**LS Mean**	**SE**	***p*** **-Value**	**LS Mean**	**SE**	***p*** **-Value**	**LS Mean**	**SE**	***p*** **-Value**	**LS Mean**	**SE**	***p*** **-Value**
Transportation				<0.001			<0.001			0.001			0.005
	Pre-transport	19.13 ^a^	0.60		16.26 ^a^	1.00		3.64 ^a^	0.56		20.29 ^a^	1.00	
	Post-transport	12.40 ^b^	0.68		25.24 ^b^	1.05		6.02 ^b^	0.82		17.50 ^b^	0.92	
Sex				0.356			0.938			0.978			0.571
	Female	16.16 ^a^	0.69		20.72 ^a^	1.05		4.69 ^a^	0.66		18.57 ^a^	0.95	
	Male	15.37 ^a^	0.68		20.78 ^a^	1.04		4.68 ^a^	0.68		19.12 ^a^	0.97	
**Random Effects**		**Exploration (s)**			**Locomotion (s)**			**Escape attempt (s)**			**Stationary (s)**		
		**Estimate**	**SE**	***p*** **-Value**	**Estimate**	**SE**	***p*** **-Value**	**Estimate**	**SE**	***p*** **-Value**	**Estimate**	**SE**	***p*** **-Value**
Facility		1.065	1.371	0.218	8.778	4.686	0.030	0.099	0.073	0.085	0.013	0.008	0.056
Dam ID (puppy ID)		21.377	6.376	0.001	11.992	6.111	0.025	0.124	0.096	0.099	0.005	0.023	0.419

^a,b^ Significant differences within traits of predictor variables; *p* < 0.01.

**Table 4:** Effect of transportation, facility, and Dam ID on focal puppy FGM (ng/g) and sIgA (mg/g) (*n* = 82).

**Fixed Effect**		**FGM (ng/g)**			**sIgA (mg/g)**		
		**LS Mean**	**SE**	***p*** **-Value**	**LS Mean**	**SE**	***p*** **-Value**
Transportation				<0.001			0.014
	Pre-transport	306.07 ^a^	42.69		2.31 ^a^	0.47	
	Post-transport	665.43 ^b^	97.73		3.55 ^b^	0.73	
**Random Effects**		**FGM (ng/g)**			**sIgA (mg/g)**		
		**Estimate**	**SE**	***p*** **-Value**	**Estimate**	**SE**	***p*** **-Value**
Facility		0.158	0.085	0.032	0.324	0.171	0.029
Dam ID		0.042	0.058	0.235	0.026	0.087	0.383

^a,b^ Significant differences within traits of predictor variables; *p* < 0.01.

**Table 5:** Effect of transportation, facility, and Dam ID on litter FGM (ng/g) and sIgA (mg/g) (*n* = 79 litters).

**Fixed Effect**		**FGM (ng/g)**			**sIgA (mg/g)**		
		**LS Mean**	**SE**	***p*-Value**	**LS Mean**	**SE**	***p*-Value**
Transportation				<0.001			0.725
	Pre-transport	330.25 ^a^	55.57		2.768 ^a^	0.482	
	Post-transport	750.57 ^b^	59.82		2.922 ^a^	0.513	
**Random Effects**		**FGM (ng/g)**			**sIgA (mg/g)**		
		**Estimate**	**SE**	***p*-Value**	**Estimate**	**SE**	***p*-Value**
Facility		2137	1337	0.055	0.187	0.117	0.056
Dam ID		1293	1258	0.152	0.195	0.133	0.072

^a,b^ Significant differences within traits of predictor variables; *p* < 0.01.

**Table 6:** Number and percentage ^1^ of puppies per each health condition pre- and post-transportation.

Health Metric *	Pre-Transportation		Post-Transportation	
	N Puppies	% Puppies	N Puppies	% Puppies
BCS-1 (thin)	1	0.28	0	0.00
BCS-2 (normal)	350	99.72	265	100.00
BCS-3 (obese)	0	0.00	0	0.00
Nasal discharge	2	0.57	2	0.75
Ocular discharge	31	8.83	45	16.98
Sneezing	1	0.28	0	0.00
Coughing	1	0.28	0	0.00
Upper Respiratory Infection (URI)	0	0.00	2	0.75
Diarrhea	1	0.28	0	0.00
Coat condition	0	0.00	1	0.38
Wound	3	0.85	0	0.00
Pyoderma	9	2.56	3	1.13
Swimmer syndrome	0	0.00	0	0.00
Tear staining—None	106	30.20	72	27.17
Tear Staining—Mild	98	27.92	103	38.87
Tear Staining—Moderate	83	23.65	63	23.77
Tear Staining—Severe	64	18.23	27	10.19

^1^ The percentages were calculated according to the real number of puppies scored during the study per each time point (*n* = 351 vs. 265, pre- and post-transportation, respectively); * Puppies can have more than one health condition.

## Data Availability

Data is available upon request to the corresponding author.

## Supplementary Materials

The following supporting information can be downloaded at: https://www.mdpi.com/article/10.3390/ani12233379/s1, Table S1. Subjects (*n* = 383 puppies; *n* = 379 puppies included in final analyses); Table S2. Inter-rater reliability (IRR) results for isolation test scoring; Table S3. Inter-rater reliability (IRR) results for puppy FIDO+ scoring; Figure S1. Descriptive statistics (mean ± SD) of variation in locomotion, escape attempt, exploration, and stationary durations among facilities pre- and post-transportation. Differences in the duration of exploration (A), escape attempt, (B) locomotion (C) and stationary behaviors (D) among facilities within and between time points (i.e., blue bars = pre-transportation: puppies of 8-week of age assessed at the kennel of origin; orange bars = post-transportation: puppies assessed 48h after arrival to the distributor) [82,83,84,85,86,87].
