# Supplementary material for: The Effect of Transportation on Puppy Welfare from Commercial Breeding Kennels to a Distributor
Source: Animals (Basel). 2022 Dec 1;12(23):3379. doi: 10.3390/ani12233379 (PMC9737031; doi:10.3390/ani12233379)
Supplement: Supplementary file 1 [file animals-12-03379-s001.zip › animals-2000785-supplementary.pdf]

Supplementary Material

Section 1:

Table S1. Subjects (*n* = 383 puppies; *n* = 379 puppies included in final analyses)

Facility	Dam ID	# of Puppies in Litter	# of Males	# of Females	Puppy Breed
1	1	5	3	2	ShihTzu
1	2	5	2	3	ShihTzu
1	3	5	5	0	MiniaturePinscher
1	4	4	1	3	MiniaturePinscher
1	5	4	1	3	Poodle/ShihTzu
1	6	3	0	3	CavalierKingCharlesSpaniel
1	7	3	2	1	ShihTzu
1	8	4	2	2	ShihTzu
2	9	4	1	3	JackRussellTerrier
2	10	5	3	2	JackRussellTerrier
2	11	8	4	4	AmericanEskimo
2	12	6	2	4	AmericanEskimo
2	13	5	1	4	JackRussellTerrier
2	14	5	4	1	JackRussellTerrier
3	15	5	4	1	ShibaInu
3	16	2	1	1	Pomeranian
3	17	1	1	0	ShetlandSheepdog
3	18	3	1	2	ShetlandSheepdog
3	19	3	1	2	ShetlandSheepdog
3	20	4	3	1	ShetlandSheepdog
4	21	5	3	2	GoldenDoodle
4	22	3	2	1	GoldenDoodle
4	23	6	3	3	GoldenDoodle
4	24	8	2	6	GoldenDoodle
4	25	10	4	6	GoldenDoodle
4	26	8	4	4	GoldenDoodle
4	27	7	1	6	GoldenDoodle
4	28	5	2	3	GoldenDoodle
5	29	3	1	2	Maltese/Poodle
5	30	5	1	4	Maltese/Yorkie

5	31	6	2	4	ToyPoodle
5	32	3	2	1	Maltese
5	33	4	2	2	Dachshund
5	34	4	3	1	Dachshund
5	35	4	2	2	Maltese
6	36	5	3	2	BichonFrise
6	37	6	3	3	Cocker/Poodle
6	38	4	3	1	Cavalier/Poodle
6	39	5	3	2	Havanese
6	40	2	2	0	Havanese
6	41	4	2	2	Havanese
6	42	5	3	2	BichonFrise
6	43	3	2	1	Havanese
6	44	6	2	4	Poodle/Bichon
7	45	6	4	2	MiniatureAustralianShepard/Poodle
7	46	2	0	2	Havanese
7	47	5	4	1	Havanese/Poodle
7	48	3	1	2	Havanese
7	49	4	3	1	BichonFrise
7	50	5	1	4	Cavalier/Bichon
8	51	4	2	2	Cocker/Poodle
8	52	4	2	2	ToyPoodle
8	53	3	1	2	MiniatureSchnauzer
8	54	5	2	3	Cavalier/Poodle
8	55	4	3	1	Pomeranian
8	56	5	2	3	Cavalier/Bichon
8	57	4	2	2	ShibaInu
8	58	1	0	1	Yorkie/Poodle
9	59	6	6	0	CavalierKingCharlesSpaniel
9	60	1	1	0	ShihTzu
9	61	2	0	2	ScottishTerrier
9	62	6	3	3	Cavalier/Bichon
9	63	4	3	1	ShihTzu
9	64	1	1	0	CairnTerrier

9	65	3	2	1	ScottishTerrier
9	66	3	2	1	CockerSpaniel
9	67	6	4	2	CavalierKingCharlesSpaniel
10	68	1	0	1	Papillion
10	69	3	1	2	Dachshund
10	70	7	5	2	Papillion
10	71	2	1	1	Dachshund
10	72	3	2	1	Papillion
10	73	2	2	0	Papillion
10	74	4	2	2	Pug
11	75	4	3	1	ToyPoodle
11	76	3	1	2	ToyPoodle
11	77	4	2	2	Poodle/ShihTzu
11	78	6	4	2	ShihTzu
11	79	3	1	2	ToyPoodle
11	80	7	4	3	ToyPoodle
12	81	5	4	1	ToyPoodle
12	82	4	0	4	Maltese
12	83	6	4	2	Maltese/ShihTzu
12	84	6	4	2	Maltese/ShihTzu
12	85	5	0	5	Cavalier/Bichon
12	86	7	1	6	ToyPoodle
12	87	6	2	4	Maltese/Yorkie
12	88	3	1	2	Maltese

Number

Section 2:

Inter-rater reliability analysis

IRR was calculated for isolation test scoring (continuous behavioral variables) using intraclass correlation coefficients (ICC) implemented by the DescTools package in R [82]. One coder (AR) scored a set of ‘gold standard’ videos. Each additional coder initially re-scored ten of those videos. If any behaviors resulted in an ICC of <0.7, coders were re-trained on scoring those behaviors and then scored an additional six videos for subsequent IRR analyses. ICC scores were interpreted using the guidelines developed by Koo & Li [83]: ≤ 0.5 = Poor, 0.5–≤0.75 = Moderate, 0.75–≤0.90 = Good, ≥ 0.90 = Excellent.

FIDO+ videos were only scored by one coder, nevertheless, IRR was also calculated to ensure accurate and reliable scoring [84]. This time, Cohen’s Kappa was used for categorical variables, implemented by the *irr* package in R [85]. Each coder scored a subset of twenty videos. Kappa values were interpreted based on the guidelines provided by McHugh [86]: 0–0.20 = None, 0.21–0.39= Minimal, 0.40–0.59 = Weak, 0.60–0.79 = Moderate, 0.80–0.90 = Strong, > 0.90 = Almost Perfect, 1 = Perfect.

IRR for each behavioral variable scored during the isolation test is reported in Table S2. ICCs for vocalization were consistently low, therefore the behavior was excluded from subsequent analyses.

IRR for FIDO+ scoring is resported in Table S3. IRR was not calculated for 'additional behaviors' due to low occurrences (see main text for description of individual behaviors).

Table S2. Inter-rater reliability (IRR) results for isolation test scoring.

Coder	Behavior	ICC3k*	p-value	95% CI	Interpretation [83]
1	Locomotion duration	0.99	0.0001	0.989, 0.990	Excellent
	Stationary duration	0.96	< 0.0001	0.959, 0.962	Excellent
	Exploration duration	0.79	0.02	0.777, 0.795	Good
	Body trembling duration	0.95[2]	0.002	0.951, 0.956	Excellent
	Frozen duration	0.85[2]	0.002	0.951, 0.956	Good
	Escape attempt duration	0.86	0.004	0.848, 0.861	Good
	Paw lift frequency	0.79	0.02	0.778, 0.796	Good
	Lip lick frequency	0.89	0.002	0.884, 0.894	Good
	Body shake frequency	1	0	-	Excellent
2	Locomotion duration	0.94	< 0.001	0.936, 0.941	Excellent
	Stationary duration	0.97	< 0.0001	0.989, 0.990	Excellent
	Exploration duration	0.94	0.0001	0.939, 0.944	Excellent
	Body trembling duration	0.95[1, 2]	0.003	0.944, 0.950	Excellent
	Frozen duration	0.95	< 0.0001	0.951, 0.955	Excellent
	Escape attempt duration	0.99[1, 2]	< 0.0001	0.994, 0.994	Excellent
	Paw lift frequency	0.74	0.03	0.729, 0.752	Moderate
	Lip lick frequency	0.89	0.002	0.884, 0.894	Good
	Body shake frequency	0.78	0.02	0.771, 0.790	Good

*ICC3k was reported as a fixed set of multiple raters scored each video [87]; [1]IRR calculated between coder 1 and 2; [2] ICC3k obtained after scoring an additional 6 videos for IRR analyses

Table S3. Inter-rater reliability (IRR) results for puppy FIDO+ scoring.

Behavior	Kappa	p-value	Interpretation [86]
Orientation	1	p < 0.0001	Perfect
Touch	1	p < 0.0001	Perfect
Response	0.76	p < 0.0001	Moderate
Modifier	0.77	p = 0	Moderate
Posture	0.60	p < 0.0001	Moderate

Section 3:

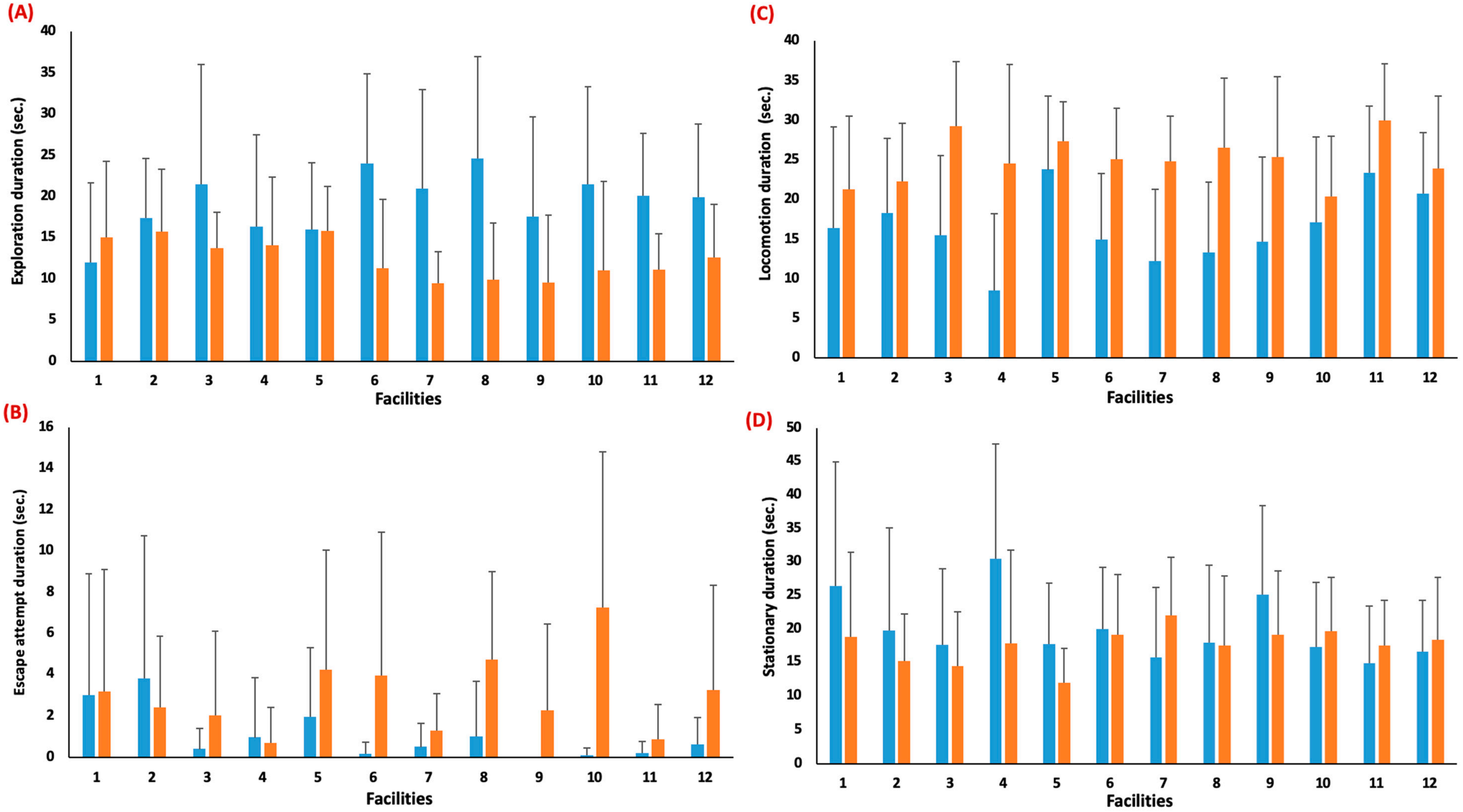

Figure S1. Descriptive statistics (mean ± SD) of variation in locomotion, escape attempt, exploration, and stationary durations among facilities pre- and post-transportation. Differences in the duration of exploration (A), escape attempt, (B) locomotion (C) and stationary behaviors (D) among facilities within and between time points (i.e., blue bars= pre-transportation: puppies of 8-week of age assessed at the kennel of origin; orange bars= post-transportation: puppies assessed 48h after arrival to the distributor).
